# Association between systemic immune-inflammation index and mortality in critically ill patients with chronic obstructive pulmonary disease: insights from the MIMIC-IV database

**DOI:** 10.3389/fmed.2025.1536652

**Published:** 2025-05-16

**Authors:** Mohan Giri, Anju Puri, Lan Huang, Shuliang Guo

**Affiliations:** ^1^Department of Respiratory and Critical Care Medicine, The First Affiliated Hospital of Chongqing Medical University, Chongqing, China; ^2^Department of Nursing, The First Affiliated Hospital of Chongqing Medical University, Chongqing, China

**Keywords:** chronic lung disease, critical care, ICU patients, immune-inflammation markers, MIMIC-IV dataset, mortality risk

## Abstract

**Background:**

Chronic Obstructive Pulmonary Disease (COPD) is a major cause of morbidity and mortality, particularly among critically ill patients. Despite the well-established role of inflammation in COPD pathogenesis, the prognostic significance of the systemic immune-inflammatory index (SII) in these patients remains unclear. This study aimed to investigate the relationship between SII and mortality risk in critically ill patients with COPD.

**Methods:**

This retrospective observational cohort study utilized data from 3,291 COPD patients extracted from the Medical Information Mart for Intensive Care-IV (MIMIC-IV 2.2) database. The participants were divided into quartiles based on their SII values. The primary endpoint was in-hospital mortality. The primary endpoint was compared across the four quartiles using Kaplan–Meier analysis. The relationship between the SII and mortality was analyzed using Cox proportional hazards models. Subgroup analyses and interaction tests were conducted to assess the robustness of the findings.

**Results:**

A total of 3,291 patients with COPD were included in the study. The in-hospital, 90-day, and 1-year mortality rates were 15.1, 27.9, and 39.4%, respectively. The results of the multivariate Cox regression analysis revealed that an elevated SII was significantly associated with in-hospital mortality (HR: 1.17; 95% CI: 1.07–1.27; *p* < 0.001), mortality at 90 days (HR: 1.26; 95% CI: 1.17–1.34; *p* < 0.001), and mortality at 1 year (HR: 1.19; 95% CI: 1.13–1.26; *p* < 0.001). Furthermore, patients in the higher quartiles of SII demonstrated an increased risk of in-hospital mortality, as well as mortality at 90 days and 1 year. The trend test across quartiles showed a statistically significant positive association between higher SII levels and increased mortality risk in all models. Stratified analysis and interaction tests demonstrated that the association between SII and in-hospital mortality remained stable.

**Conclusion:**

Our study demonstrates that a high SII is independently associated with an increased risk of mortality in critically ill COPD patients. SII may serve as a risk stratification and prognostication tool in patients with COPD. Larger prospective studies are needed to validate these findings.

## 1 Introduction

Chronic obstructive pulmonary disease (COPD) is a prevalent and burdensome respiratory illness that imposes a substantial strain on both individuals and healthcare systems globally (1). COPD was a leading cause of global mortality, with 212.3 million cases and 3.3 million deaths reported in 2019 (2). The global burden of COPD is projected to increase by 23% from 2020 to 2050, reaching nearly 600 million cases by 2050 (1). Given the progressive nature of COPD and its substantial impact on quality of life, early identification of at-risk patients and effective clinical management are crucial. Studies have demonstrated that hematologic inflammatory biomarkers obtained from routine blood tests, such as the neutrophil-percentage-to-albumin ratio (NPAR), the platelet-to-lymphocyte ratio (PLR), and the neutrophil-to-lymphocyte ratio (NLR), have shown significant associations with mortality in patients with COPD (3, 4). There remains an unmet need for the identification of simple, convenient, and effective biomarkers to predict the prognosis of COPD, guide clinical decision-making, and identify patients at risk of mortality.

Systemic inflammation is a hallmark of COPD, particularly in critically ill patients, and becomes more pronounced during exacerbations (5). This is evidenced by increased levels of circulating cytokines, chemokines, and acute-phase proteins, along with significant alterations in circulating cell populations, such as monocytes, neutrophils, and lymphocytes (5–7). The systemic immune-inflammation index (SII), a novel and comprehensive inflammatory biomarker derived from absolute platelet, neutrophil, and lymphocyte counts, is a stable and reliable indicator of both localized and systemic immune responses and overall systemic inflammation. In recent years, SII has been extensively used as a novel prognostic systemic inflammatory biomarker for various diseases, including hepatocellular carcinoma (8, 9), metabolic syndrome (10), asthma (11), coronary artery disease (12), psoriasis (13), stroke-associated pneumonia (14), chronic kidney disease (15), and COVID-19 (16). SII is a promising biomarker for predicting the severity, prognosis, and risk of complications in patients with COPD (17). Elevated SII levels are strongly associated with increased COPD severity, impaired lung function, and a higher risk of mortality (17–21). However, the relationship between SII and mortality in COPD, especially among critically ill patients, remains unclear. Further research is needed to confirm these associations and better define SII’s clinical utility in managing and treating COPD patients.

The present study aims to investigate the association between SII and all-cause mortality (ACM) in critically ill COPD patients using the Medical Information Mart for Intensive Care III (MIMIC-IV) database. The findings of this study could open new avenues for improving patient outcomes by identifying high-risk individuals with COPD and enabling timely interventions, thereby addressing a critical gap in both current clinical practice and research.

## 2 Materials and methods

### 2.1 Data source

This retrospective observational analysis used data from the Medical Information Mart for Intensive Care IV (MIMIC-IV version 2.2) database. MIMIC-IV is an extensive, open-access public database containing over 73,000 ICU admissions to the Beth Israel Deaconess Medical Center in Boston, Massachusetts, from 2008 to 2019 (22). One author (MG) completed the training course ‘CITI Data or Specimens Only Research’ and was authorized to access the MIMIC-IV database for data extraction (certification number: 45355193). This study was conducted following the ethical principles of the Helsinki Declaration. The institutional review boards of the Massachusetts Institute of Technology and the Beth Israel Deaconess Medical Center have approved using this database for research. Due to the de-identified nature of the data, the requirement for informed consent was waived for this study.

### 2.2 Study population

**Figure 1:**
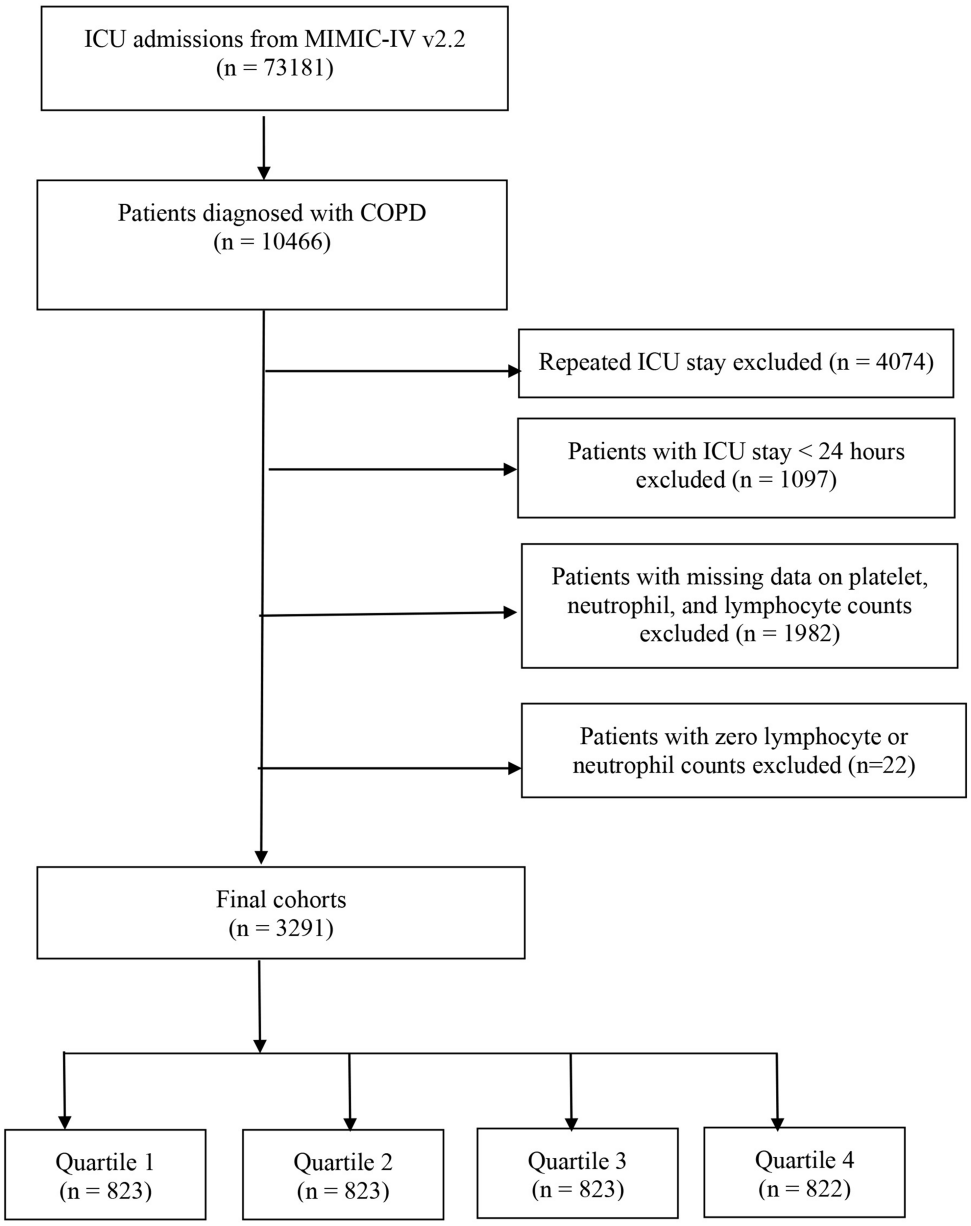
Flowchart of patient selection process.

This study enrolled all patients aged ≥ 18 years diagnosed with COPD according to the International Classification of Diseases, 9th and 10th revision (ICD-9 and ICD-10) codes. Supplementary Table 1 includes the ICD codes employed to identify patients with COPD. The exclusion criteria included: (i) patients who were under 18 years old; (ii) individuals with multiple ICU admissions for COPD; (iii) those admitted to the ICU for less than 24 h; and (iv) Patients with data on platelets, neutrophils, and lymphocyte counts missing or zero. The study population comprised 3,291 patients, who were subsequently classified into four groups based on the quartiles of their SII values, as measured on their first day of ICU admission (Figure 1).

### 2.3 Data extraction

Data extraction was performed using the Structured Query Language (SQL) with script codes obtained from the GitHub repository^1^. The following variables were extracted: age, sex, length of hospital stay, length of ICU stay, hospital death status, and the Charlson Comorbidity Index (CCI). The severity of illness scores at admission included the Simplified Acute Physiology Score II (SAPS-II), Oxford Acute Severity of Illness Score (OASIS), and the Sepsis-related Organ Failure Assessment (SOFA) score. Laboratory parameters encompassed hemoglobin (Hb), white blood cell count (WBC), red blood cell count (RBC), platelet count, lymphocyte count (LYM), neutrophil count (NEU), glucose, serum creatinine, blood urea nitrogen (BUN), bicarbonate, international normalized ratio (INR), and activated partial thromboplastin time (APTT). In addition, comorbidities such as hypertension, diabetes mellitus, congestive heart failure, coronary artery disease (CAD), renal disease, malignant cancer, severe liver disease, and cerebrovascular disease were documented. The extracted data also included vital signs, such as heart rate, respiratory rate, mean arterial pressure (MAP), and peripheral oxygen saturation (SpO2). Additionally, information on the need for mechanical ventilation, renal replacement therapy (RRT), or the use of diuretics was recorded. The Systemic Inflammatory Index (SII) was calculated using the formula: Ln [(neutrophil count × platelet count)/lymphocyte count]. All laboratory variables were obtained within the first 24 h following patient admission. The average value was used for laboratory measurements taken multiple times within 24 h. We excluded variables with over 20% missing values to reduce potential bias. For variables with missing data below 20%, a random forest method for multiple imputations using the ‘mice’ package in R was utilized (23).

### 2.4 Primary outcome and secondary outcomes

The primary endpoint of this study was in-hospital mortality, and the secondary endpoint encompassed mortality within 90 days and 1 year after admission to the ICU.

### 2.5 Statistical analysis

This study categorized participants into quartiles (Q1–Q4) based on their SII values. The normality of continuous variables was assessed using the Kolmogorov–Smirnov test. Continuous variables were reported as the mean ± standard deviation (SD) for normally distributed data, while non-normally distributed data were presented as the median and interquartile range (IQR). Analysis of variance (ANOVA) or *t*-tests were used for continuous variables that were normally distributed, whereas the Mann–Whitney *U* test or Kruskal–Wallis test was utilized for non-normally distributed variables. The categorical variables were analyzed using Fisher’s exact or chi-square tests, with results presented as counts (percentages). Kaplan–Meier survival analysis was used to analyze the incidence of the endpoint in various groups stratified by SII levels, and the log-rank test was employed to assess differences in survival between these groups. The relationship between SII and the study endpoints was quantified using Cox proportional hazards models to calculate hazard ratios (HRs) and 95% confidence intervals (CIs). Univariable Cox regression analysis was conducted to examine the association between various factors and in-hospital mortality in the study population. Clinically significant variables and those with a *p* < 0.05 in the univariate analysis were incorporated into the multivariable Cox proportional hazards model. The analysis included three models for confounder adjustment: Model 1 (unadjusted model), Model 2 (adjusted for age and gender), and Model 3 (adjusted for age, gender, SOFA score, hemoglobin, WBC, platelet count, glucose levels, hypertension, diabetes, coronary artery disease, malignant cancer, severe liver disease, and mechanical ventilation). The SII was included in the models as both continuous and categorical variables, with the first quartile of the SII serving as the reference category. Moreover, stratified analyses were performed based on age (≤65 and >65 years), gender, hypertension, diabetes, congestive heart failure, coronary artery disease, renal failure, malignant cancer, severe liver disease, cerebrovascular disease, and mechanical ventilation to assess the consistency of the SII’s prognostic value for the primary outcome. All statistical analyses were carried out using R version 4.4.1 (R Foundation), with a two-sided *p*-value of less than 0.05 deemed statistically significant.

## 3 Results

This study included 3,291 critically ill patients diagnosed with chronic obstructive pulmonary disease (COPD). The cohort’s median age was 72 years (IQR: 64.0–80), with males comprising 54.6% of the population. The median SII was 7.4 (6.7–8.2). The in-hospital mortality was 15.1% (498/3,291). The 90-day and 1-year all-cause mortality rates were 27.9 and 39.4%, respectively (Table 1).

**Table 1:** Baseline characteristics of COPD patients across quartiles of the Systemic Immune-Inflammation Index (SII).

Characteristics	Overall (*N* = 3,291)	Q1 (*N* = 823)	Q2 (*N* = 823)	Q3 (*N* = 823)	Q4 (*N* = 822)	*P*-value
Age (Y, IQR)	72 (64–80)	70 (62–79)	71 (63–81)	73 (65–81)	73 (65–81)	<0.001
Gender, Male (*N*, %)	1797 (54.6)	492 (59.8%)	469 (57.0%)	430 (52.2%)	406 (49.4%)	<0.001
SII	7.4 (6.7–8.2)	6.2 (5.8–6.5)	7.1 (6.9–7.2)	7.8 (7.58–8)	8.7 (8.42–9.07)	<0.001
CCI	6 (5–8)	6 (4–8)	6 (4.5–8)	6 (5–8)	6 (5–8)	<0.001
Clinical severity						
SOFA	5 (3–7)	5 (3–7)	4 (2–7)	5 (2–7)	5 (3–7)	0.005
SAPS-II	38 (31–47)	37 (29.5–45)	37 (31–45)	38 (31–48)	40 (33–50)	<0.001
OASIS	33 (28–39)	31 (27–37)	33 (28–38)	34 (28–39)	35 (29–42)	<0.001
Laboratory parameters						
Hemoglobin, G/Dl	10.6 (9.3–12.2)	10.4 (9.1–12)	10.8 (9.3–12.5)	10.8 (9.4–12.3)	10.5 (9.25–11.94)	<0.001
WBC, 109/L	11.6 (8.5–15.8)	9.2 (6.6–12.7)	10.5 (8.1–13.7)	12 (9.07–15.3)	15.7 (11.68–20.15)	<0.001
RBC, 109/L	3.6 (3.1–4.1)	3.4 (3–4)	3.6 (3.1–4.2)	3.6 (3.16–4.2)	3.6 (3.15–4.1)	<0.001
Platelets, 109/L	194 (141.2–260)	138.3 (104.7–180.9)	181.2 (141.7–227.3)	209 (163.42–265.8)	272.17 (205–359)	<0.001
Neutrophils, 109/L	9.3 (6.3–13.6)	5.8 (4.1–8.2)	8.3 (6.1–11)	10.2 (7.8–13.6)	14.5 (10.8–18.9)	<0.001
Lymphocytes, 109/L	1.1 (0.6–1.7)	1.8 (1.2–2.7)	1.3 (0.9–1.8)	0.9 (0.63–1.3)	0.6 (0.38–0.89)	<0.001
Glucose, Mg/Dl	130 (109–164.8)	119.5 (103–145.2)	125.5 (106.5–159.8)	135.5 (113–172)	144.5 (117.62–178.5)	<0.001
Creatinine, Ng/Dl	1 (0.8–1.6)	1 (0.8–1.4)	1 (0.8–1.4)	1.1 (0.75–1.8)	1.05 (0.75–1.65)	0.005
BUN, Mg/Dl	22.5 (15–36)	19.5 (14–30)	21 (15–32.5)	24 (16–39.8)	25.5 (17–40.88)	<0.001
Bicarbonate, Meq/L	23.5 (21–26.5)	23.5 (21.5–25.5)	24 (21.5–26.5)	24 (21–27)	24 (21–27.5)	0.015
INR	1.2 (1.1–1.4)	1.2 (1.1–1.4)	1.2 (1.1–1.4)	1.2 (1.1–1.5)	1.25 (1.1–1.5)	0.544
APTT (S)	31.2 (27.6–38.9)	31.1 (28–37.7)	31.4 (27.7–40)	31.4 (27.6–40.1)	30.85 (27.16–38.84)	0.378
Comorbidities						
Hypertension	1,374 (41.8)	342 (41.6%)	338 (41.1%)	354 (43.0%)	340 (41.4%)	0.859
Diabetes	1,087 (33.0)	261 (31.7%)	279 (33.9%)	279 (33.9%)	268 (32.6%)	0.735
Congestive heart failure	1,419 (43.1)	303 (36.8%)	352 (42.8%)	389 (47.3%)	375 (45.6%)	<0.001
Coronary artery disease	1,106 (33.6)	292 (35.5%)	282 (34.3%)	271 (32.9%)	261 (31.8%)	0.409
Renal disease	821 (24.9)	186 (22.6)	208 (25.3)	215 (26.1)	212 (25.8)	0.335
Malignant cancer	499 (15.2)	112 (13.6)	96 (11.7)	122 (14.8)	169 (20.6)	<0.001
Severe liver disease	120 (3.6)	49 (6)	40 (4.9)	20 (2.4)	11 (1.3)	<0.001
Cerebrovascular disease	475 (14.4)	147 (17.9)	121 (14.7)	123 (14.9)	84 (10.2)	<0.001
Monitoring parameters						
Heart Rate, Bpm	84.6 (75.4–96.4)	81.3 (73.9–91.5)	83 (74.3–93.4)	85.1 (75.45–97.1)	90.5 (78.93–100.44)	<0.001
MAP, Mmhg	75.2 (69.6–82.2)	75.5 (70.1–82.1)	75 (69.8–82.4)	75.2 (69.22–82.3)	74.3 (69.08–81.56)	0.168
RR, Breaths/Minutes	19.6 (17.3–22.3)	18.9 (16.8–21.5)	18.9 (17–21.7)	20 (17.65–22.6)	20.5 (18.31–23.47)	<0.001
Spo2, %	96.4 (94.8–97.8)	96.8 (95.2–98)	96.5 (94.9–97.9)	96.1 (94.42–97.6)	96.1 (94.56–97.58)	<0.001
Intervention						
Mechanical ventilation	1,530 (46.5)	388 (47.1)	390 (47.4)	366 (44.5)	386 (47.0)	0.609
Diuretics	898 (27.3)	190 (23.1)	227 (27.6)	251 (30.5)	230 (28.0)	0.008
RRT	234 (7.1)	54 (6.6)	51 (6.2)	73 (8.9)	56 (6.8)	0.146
Events						
LOS ICU, days	2.8 (1.7–5.3)	2.4 (1.5–4.3)	2.7 (1.7–5.1)	3 (1.75–5.4)	3.31 (1.88–6.7)	<0.001
LOS hospital, days	8 (5–13)	7 (5–12)	8 (5–13)	8 (5–13)	9 (5–15)	<0.001
In-hospital mortality	498 (15.1)	88 (10.7)	88 (10.7)	143 (17.4)	179 (21.8)	<0.001
90 days mortality	917 (27.9)	160 (19.4)	167 (20.3)	253 (30.7)	337 (41.0)	<0.001
1 year mortality	1,297 (39.4)	245 (29.8)	264 (32.1)	358 (43.5)	430 (52.3)	<0.001

Continuous variables are presented as median (interquartile range), while categorical variables are shown as *N* (%). Q1–Q4 refers to groups categorized by quartiles based on the SII. APTT, activated partial thromboplastin time; BUN, blood urea nitrogen; CCI, Charlson comorbidity index; CAD, coronary artery disease; INR, international normalized ratio; LOS ICU, length of stay in the intensive care unit; MAP, mean arterial pressure; RBC, red blood cell count; RR, respiratory rate; RRT, renal replacement therapy; SII, systemic immune-inflammation index; SOFA, sequential organ failure assessment; SAPSII, simplified acute physiology score II; Spo2, peripheral capillary oxygen saturation; WBC, white blood cell count.

### 3.1 Baseline characteristics

Table 1 presents the baseline demographic and clinical characteristics, categorized by quartiles of the log-transformed SII values. The median SII values by quartile were 6.2 (IQR: 5.8–6.5) for Q1, 7.1 (IQR: 6.9–7.2) for Q2, 7.8 (IQR: 7.58–8.0) for Q3, and 8.7 (IQR: 8.42–9.07) for Q4. Patients in the higher quartiles of the SII were older and exhibited more severe clinical characteristics, as evidenced by higher SOFA, SAPS-II, and OASIS scores (all *p*-value < 0.001). The analysis revealed statistically significant differences in the distribution of gender, CCI, hemoglobin, WBC, RBC, platelets, neutrophils, lymphocytes, glucose, creatinine, BUN, bicarbonate, history of congestive heart failure, malignant cancer, severe liver disease, cerebrovascular disease, diuretic use, heart rate, respiratory rate, and SpO2 across all quartiles of SII (*p* < 0.05). No significant differences were found between the different quartiles regarding INR, APTT, mean arterial pressure, history of hypertension, diabetes, coronary artery disease, renal disease, and the use of renal replacement therapy (RRT) and mechanical ventilation (all *p* > 0.05). Furthermore, patients in higher quartiles of SII exhibited longer lengths of stay in both the ICU and hospital, along with higher rates of in-hospital mortality and all-cause mortality at 90 days and 1 year compared to those in lower quartiles (Table 1).

### 3.2 Associations of SII with in-hospital mortality, 90-day mortality, and 1-year mortality

The relationship between SII and in-hospital mortality was investigated using univariate Cox proportional hazards regression analysis, which revealed that significant variables affecting in-hospital mortality include age, CCI, SOFA, SAPS, OASIS, laboratory values such as WBC, glucose, creatinine, BUN, bicarbonate, and INR, as well as comorbidities such as hypertension, diabetes, malignancy, severe liver disease, and the need for mechanical ventilation or renal replacement therapy (Supplementary Table 2). When the SII was considered as a continuous variable, Cox proportional hazards analysis indicated that SII is an independent predictor of in-hospital mortality across all models: unadjusted Model 1 (HR: 1.16; 95% CI: 1.08–1.25; *p* < 0.001), Model 2 (HR: 1.13; 95% CI: 1.05–1.22; *p* < 0.001), and fully adjusted Model 3 (HR: 1.17; 95% CI: 1.07–1.27; *p* < 0.001) (Table 2). Moreover, when treated as a continuous variable, SII is an independent predictor of 90-day and 1-year all-cause mortality across all three models (Model 1, Model 2, and Model 3). The detailed associations between SII and all-cause mortality at 90 days and 1 year are presented in Table 2.

**Table 2:** Association of the SII with in-hospital, 90-day, and 1-year mortality in patients with COPD.

Categories	Model 1	*P-*value	Model 2	*P-*value	Model 3	*P*-value
	HR (95% CI)		HR (95% CI)		HR (95% CI)	
In-hospital mortality						
SII (continuous variable)	1.16 (1.08–1.25)	<0.001	1.13 (1.05–1.22)	0.001	1.17 (1.07–1.27)	<0.001
SII quartiles						
Q1	Reference		Reference		Reference	
Q2	0.99 (0.74–1.33)	>0.9	0.93 (0.69–1.25)	0.6	0.95 (0.70–1.29)	0.7
Q3	1.61 (1.23–2.10)	<0.001	1.52 (1.17–1.99)	0.002	1.70 (1.28–2.25)	<0.001
Q4	1.72 (1.33–2.22)	<0.001	1.56 (1.20–2.01)	<0.001	1.68 (1.25–2.27)	<0.001
*P* for trend	<0.001		<0.001		<0.001	
90 days mortality						
SII (continuous variable)	1.31 (1.24–1.39)	<0.001	1.28 (1.21–1.36)	<0.001	1.26 (1.17–1.34)	<0.001
SII Quartiles						
Q1	Reference		Reference		Reference	
Q2	1.04 (0.84–1.30)	0.7	1.00 (0.80–1.24)	>0.9	1.05 (0.84–1.31)	0.7
Q3	1.71 (1.41–2.09)	<0.001	1.63 (1.34–1.99)	<0.001	1.78 (1.45–2.20)	<0.001
Q4	2.39 (1.98–2.88)	<0.001	2.23 (1.84–2.69)	<0.001	2.20 (1.77–2.75)	<0.001
*P* for trend	<0.001		<0.001		<0.001	
1-year mortality						
SII (continuous variable)	1.25 (1.19–1.31)	<0.001	1.22 (1.16–1.29)	<0.001	1.19 (1.13–1.26)	<0.001
SII quartiles						
Q1	Reference		Reference		Reference	
Q2	1.09 (0.91–1.29)	0.3	1.05 (0.88–1.25)	0.6	1.10 (0.92–1.31)	0.3
Q3	1.64 (1.39–1.93)	<0.001	1.57 (1.33–1.84)	<0.001	1.66 (1.40–1.98)	<0.001
Q4	2.13 (1.82–2.49)	<0.001	2.01 (1.72–2.35)	<0.001	1.94 (1.61–2.34)	<0.001
*P* for trend	<0.001		<0.001		<0.001	

Model 1 was not adjusted.

Model 2 was adjusted for age and gender.

Model 3 was adjusted for age, gender, SOFA score, hemoglobin, white blood cell count, platelet count, glucose levels, hypertension, diabetes, coronary artery disease, malignant cancer, severe liver disease, and mechanical ventilation.

Individuals in the highest quartile (Q4) of the SII demonstrated a significantly increased risk of in-hospital mortality across all models when SII was treated as a categorical variable: Model 1 (HR: 1.72; 95% CI: 1.33–2.22; *p* < 0.001), Model 2 (HR: 1.56; 95% CI: 1.20–2.01; *p* < 0.001), and Model 3 (HR: 1.68; 95% CI: 1.25–2.27; *p* < 0.001) (Table 2). The trend test evaluating the association between SII and in-hospital mortality was statistically significant across all three models (*P* for trend < 0.001), indicating a linear and positive relationship between SII levels and in-hospital mortality, particularly evident in the highest quartiles (Q3 and Q4).

For 90-day all-cause mortality, patients in the highest quartile (Q4) of the SII demonstrated a markedly elevated risk across all models when analyzed as a categorical variable: Model 1 (HR: 2.39; 95% CI: 1.98–2.88; *p* < 0.001), Model 2 (HR: 2.23; 95% CI: 1.84–2.69; *p* < 0.001), and Model 3 (HR: 2.20; 95% CI: 1.77–2.75; *p* < 0.001) (Table 2). The trend test also indicated a statistically significant correlation between SII and 90-day mortality (*P* for trend < 0.001), suggesting a linear and positive association. The multivariate Cox regression analyses of SII as a categorical variable and 1-year mortality also showed similar results, and Table 2 summarizes the detailed associations.

### 3.3 Kaplan–Meier survival curve

**Figure 2:**
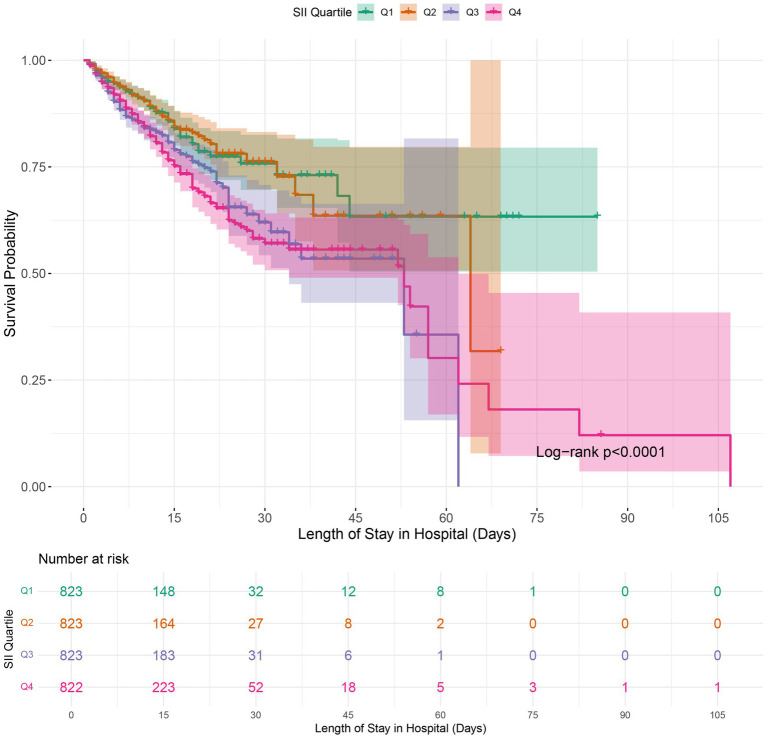
Kaplan–Meier survival analysis for in-hospital mortality in COPD patients based on SII quartile groups. SII, Systemic Immune-Inflammation Index.

**Figure 3:**
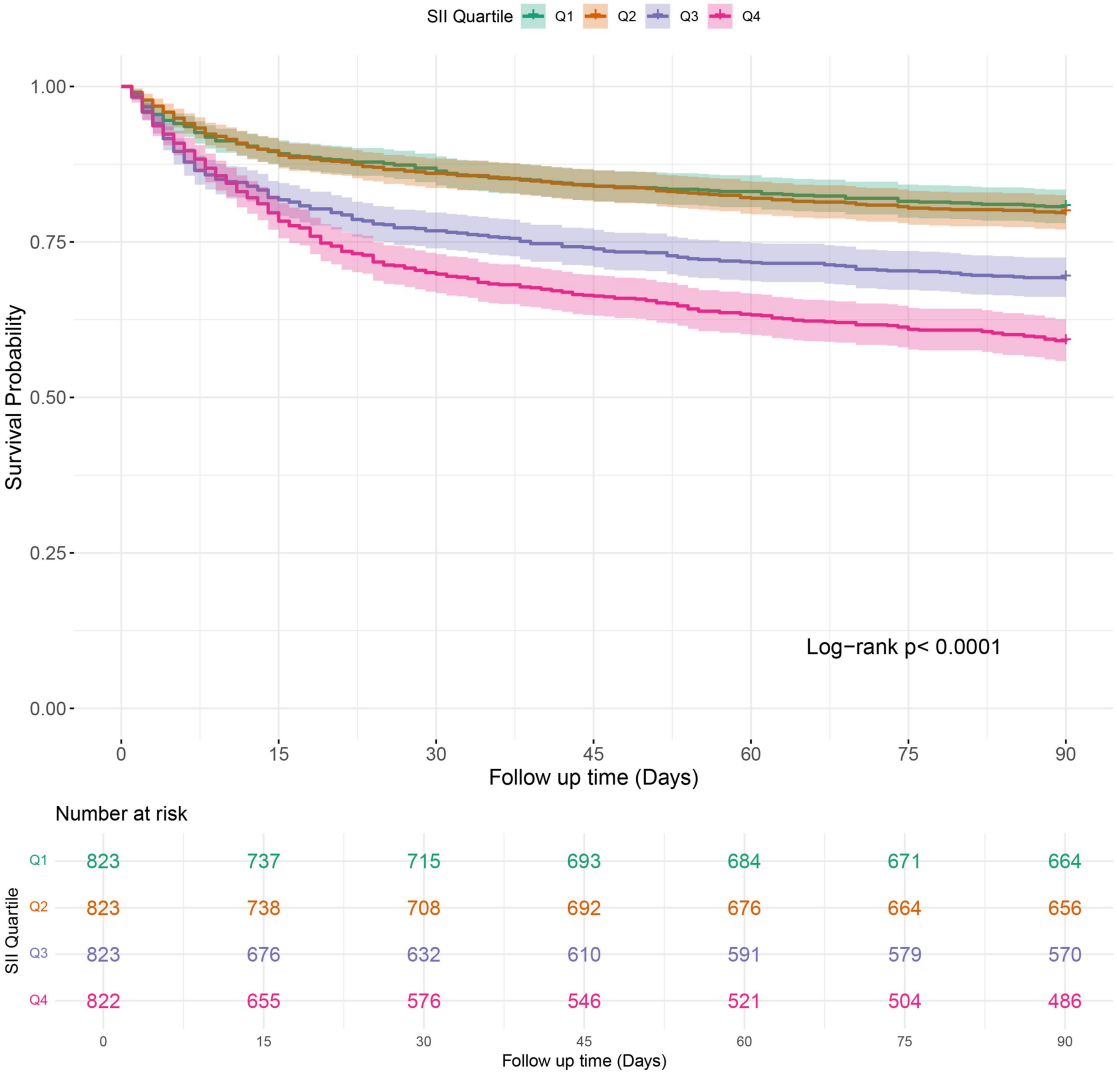
Kaplan–Meier survival analysis of 90-days mortality in COPD patients stratified by SII quartile groups. SII: Systemic Immune-Inflammation Index.

**Figure 4:**
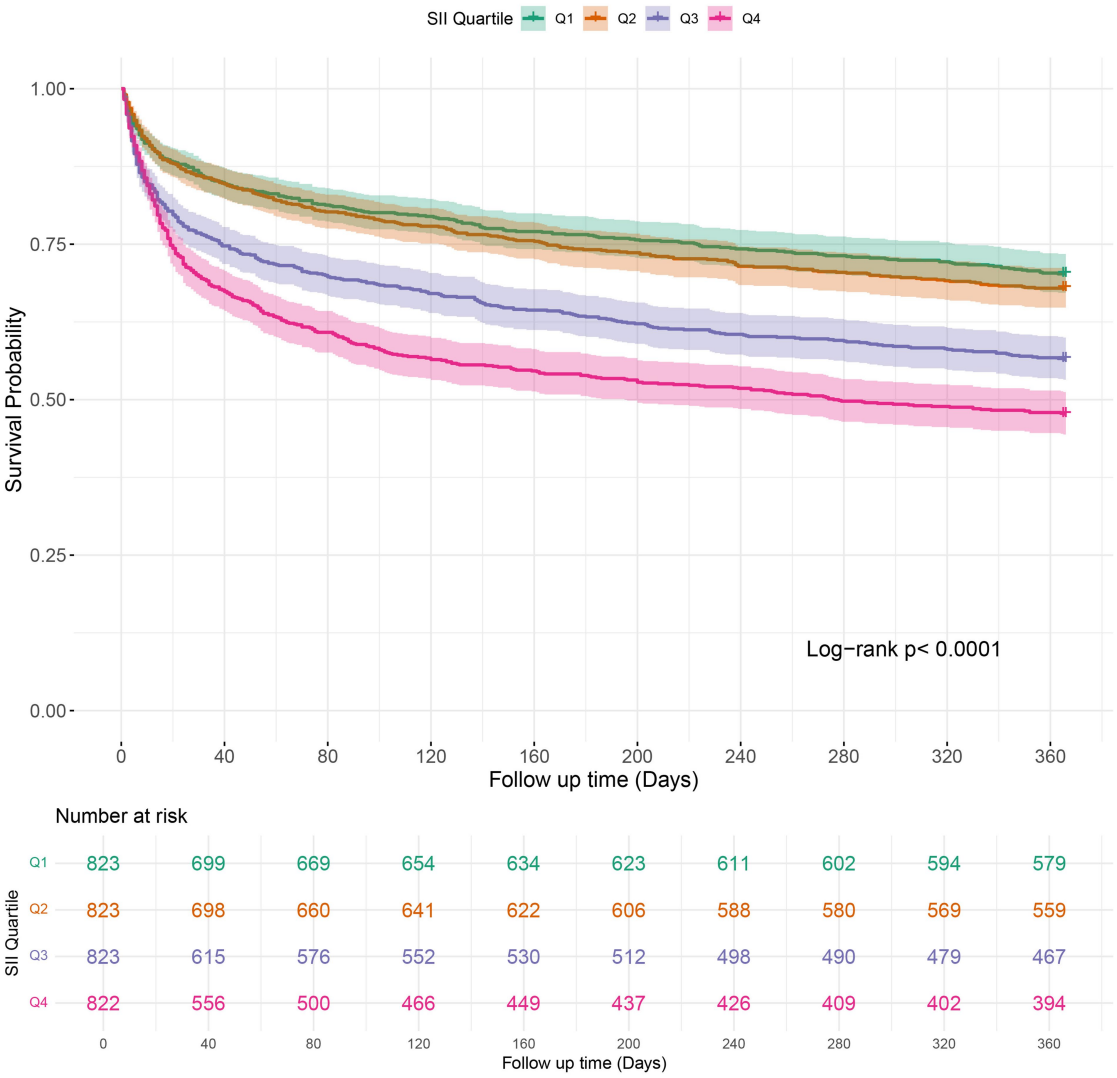
Kaplan–Meier survival analysis curves for 1-year mortality in patients with COPD based on SII quartiles. SII: Systemic Immune-Inflammation Index.

Kaplan–Meier survival analysis was employed to analyze in-hospital mortality, 90-day, and 1-year all-cause mortality rates across various SII quartiles. The results indicated that individuals in the highest quartile (Q4) had a higher in-hospital mortality rate, with a hazard ratio of 1.7018 (95% CI: 1.3326 to 2.1733) compared to the reference group (Q1). Furthermore, the Log-rank test revealed a statistically significant difference in survival across the quartiles, with a log-rank *p*-value of < 0.0001 (Figure 2). Moreover, Kaplan–Meier analysis showed a statistically significant difference in all-cause mortality rates between patients in the highest SII quartile and those in the lower quartiles at 90 days and 1 year (log-rank *p* < 0.0001 for both comparisons) (Figures 3, 4).

### 3.4 Subgroup analysis

**Figure 5:**
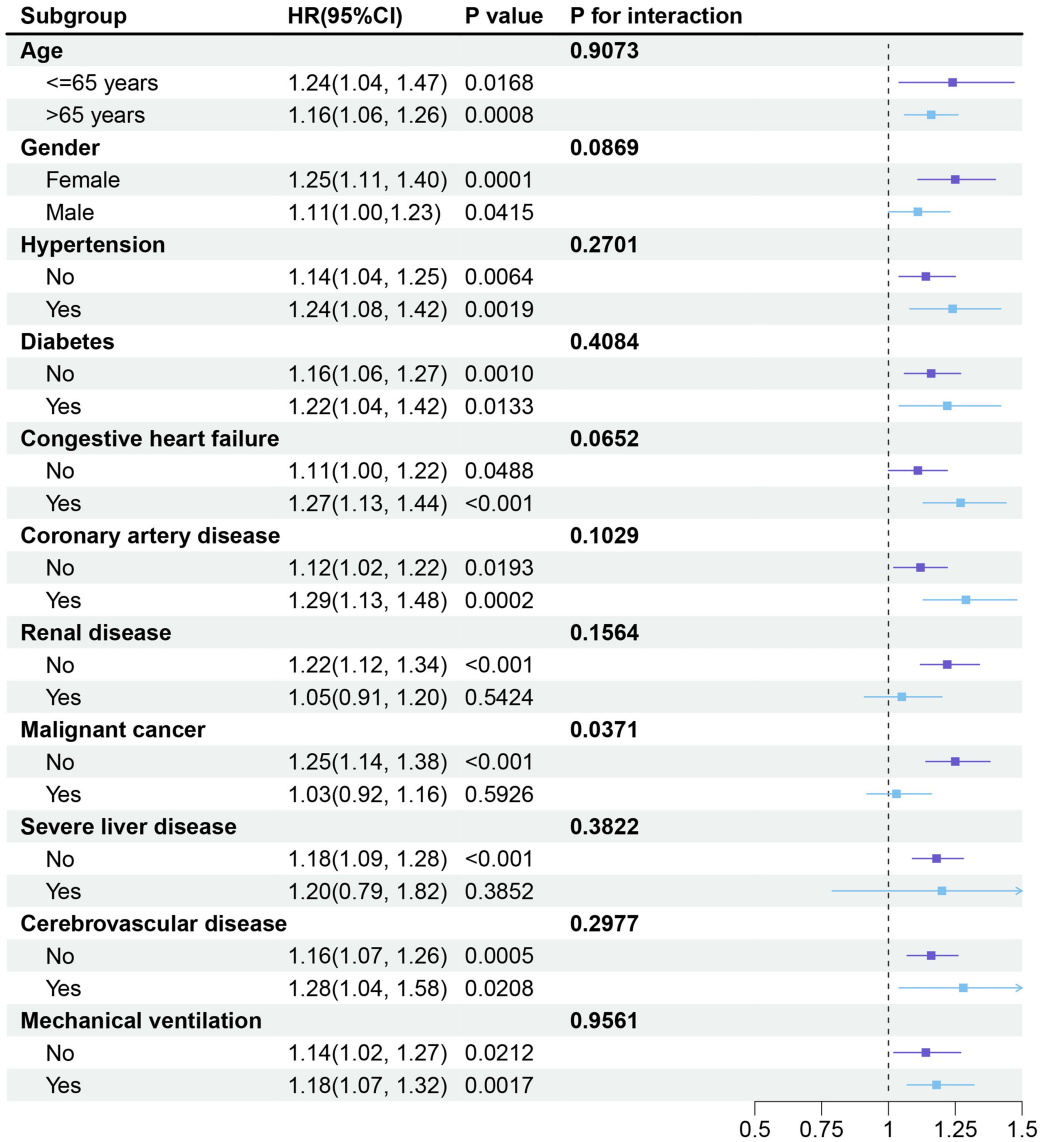
Forest plot depicting the subgroup analysis of the association between SII and in-hospital mortality in critically ill patients with COPD. The stratified analyses were adjusted for all covariates, except for the stratification variable. HR: Hazard ratio; SII: Systemic Immune-Inflammation Index.

Furthermore, to confirm the relationship between SII and the primary endpoint of the study (in-hospital mortality), we conducted subgroup analyses based on age, gender, hypertension, diabetes, congestive heart failure, coronary artery disease, renal disease, malignant cancer, severe liver disease, cerebrovascular disease, and mechanical ventilation. SII was significantly associated with a higher risk of in-hospital mortality in COPD patients across all subgroups, except for those with renal disease (HR: 1.05; 95% CI: 0.91–1.20; *p* > 0.05), malignant cancer (HR: 1.03; 95% CI: 0.92–1.16; *p* > 0.05), and severe liver disease (HR: 1.20; 95% CI: 0.79–1.82; *p* > 0.05) (Figure 5). Additionally, all stratified analyses revealed no interaction between the variables and the SII index (all *p*-values for interaction > 0.05).

## 4 Discussion

To our knowledge, this is the largest and most comprehensive cohort study to date examining the relationship between SII levels and in-hospital and all-cause mortality among critically ill COPD patients. We demonstrated that higher SII levels were independently associated with an increased risk of in-hospital and all-cause mortality in this patient population. Even after adjusting for the potential confounding risk factors, this association remained significant, underscoring our findings’ robustness. Moreover, Kaplan–Meier survival curves indicated that COPD patients in the highest SII quartile (Q4) exhibited a higher risk of hospital as well as all-cause mortality compared to those in the lower quartiles (Q1). The subgroup analysis revealed no significant interaction between subgroups and in-hospital mortality. Therefore, SII could serve as a novel and promising biomarker for identifying critically ill COPD patients at elevated risk of mortality in hospital settings.

The SII has recently been proposed as a potential marker for multiple diseases, including metabolic disorders, cardiovascular disease, and respiratory diseases (10–12). A population-based study (19) that examined the association between sarcopenia, SII, and mortality in individuals with and without COPD or asthma found that elevated SII levels were linked to increased mortality risk, regardless of COPD status. This suggests that systemic inflammation, as evidenced by elevated SII levels, is a significant risk factor for mortality, particularly in combination with sarcopenia. Gao et al. (24) demonstrated that higher SII values can be used to predict patients at greater risk for readmission in acute exacerbations of bronchiectasis, highlighting SII’s role as a useful prognostic marker. Another study (20) investigating the association between inflammatory markers, including the SII and leukocyte-albumin ratio, and the comorbidity of COPD and lung cancer found that elevated levels of these markers were independent risk factors for lung cancer comorbid with COPD. A study (25) based on data from adults aged 40 years and above in the United States demonstrated a significant positive association between elevated SII levels and COPD risk, suggesting that SII could be a potential biomarker for identifying individuals at elevated risk of COPD. Furthermore, SII exhibited slightly better predictive performance than other inflammatory markers, such as NLR and PLR, highlighting its potential clinical utility. In a study of 1,653 critically ill COPD patients, Zhang et al. (18) observed that higher SII levels were linked to an increased risk of respiratory failure in-hospital and long-term mortality. These studies collectively indicate a potential association between the SII and clinical outcomes in patients with COPD. Our findings align with previous studies suggesting that SII is an independent predictor of mortality in COPD patients. However, the relationship between SII and mortality risk may vary depending on the severity of COPD and the different stages of the disease. As the disease progresses, immune-inflammatory responses tend to increase, which could lead to differences in how SII functions across various stages of COPD. In more advanced COPD, increased systemic inflammation, resulting from chronic tissue damage, oxidative stress, and comorbidities, might cause SII to behave differently than in earlier stages.

The precise mechanisms underlying the association between higher SII and poor prognosis in COPD patients are yet to be elucidated. SII, derived from neutrophil, platelet, and lymphocyte counts, indicates a state of systemic inflammation. Systemic alterations in neutrophils play a vital role in the pathogenesis of COPD and contribute to poor prognosis by exacerbating inflammation, causing tissue damage, increasing the frequency and severity of exacerbations, and promoting the development of comorbidities (26). Neutrophils are the primary immune cells mobilized during inflammation, secreting proteases that lead to airway injury (26). This mechanism activates group 3 innate lymphoid cells, enhancing the inflammatory response. Moreover, hyperactivated neutrophils release proinflammatory cytokines, such as interleukin-8 (IL-8) and TNF-*α*, further exacerbating inflammation (27). Oxidative stress, induced by an imbalance of oxidants and antioxidants, further stimulates immune cells, increasing the production of reactive oxygen species. This interplay between inflammation and oxidative stress contributes to progressive lung tissue damage, perpetuating a structural and functional decline cycle (27). Enhanced platelet aggregability can mediate the lungs’ thrombotic, inflammatory, and immune processes, potentially accelerating the progression of COPD (28). Elevated platelet counts are associated with higher mortality and cardiovascular morbidity due to systemic inflammation, plaque destabilization, and platelet activation (29). Fibrinogen, a biomarker tied to COPD mortality, is linked to platelet count and regulated by interleukin-6, a pro-inflammatory cytokine that increases thrombopoietin levels, driving platelet production (30). Activated platelets interact with endothelial cells, and leukocytes play a crucial role in inflammatory response and immune regulation (31). Lymphocytes play a crucial role in COPD pathogenesis. Increased numbers of CD8 + T cells correlate with alveolar destruction and airflow limitation (32). CD4 + T cells, particularly TH1 and TH17 subsets, contribute to neutrophilic inflammation by releasing pro-inflammatory cytokines like IL-17A and IL-22 (32, 33). Chemokine signaling, primarily via CXCR3, promotes lung lymphocyte recruitment, exacerbating inflammation (34). Moreover, chronic infections in COPD patients can perpetuate T-cell-driven immune responses, exacerbating airway inflammation and contributing to disease progression. These persistent immune reactions impair lung function and increase the risk of acute exacerbations. Chronic lung inflammation in COPD is characterized by the infiltration of immune cells, including alveolar macrophages, neutrophils, and T lymphocytes, along with the generation of proinflammatory mediators (35). This continuous inflammatory cycle leads to airway remodeling, tissue damage, and a decline in pulmonary function, all of which contribute to the poor prognosis and increased mortality risk in COPD patients.

This study offers several notable strengths. This is the most comprehensive study to date because it includes a significantly larger cohort of 3,291 patients compared to the previous research, enabling a more precise evaluation of the association between SII and mortality in Patients with COPD. Furthermore, the extended follow-up period and rigorous adjustment for confounding variables contribute to a more comprehensive understanding of SII’s role in predicting patient mortality. Given the routine measurement of neutrophils, platelets, and lymphocytes in blood tests, the SII provides an easily accessible and practical index for risk assessment in critically ill COPD patients. This research suggests clinicians can identify individuals at higher risk for adverse outcomes by utilizing this cost-effective biomarker.

Nevertheless, this study has certain limitations. First, the retrospective nature of this study limits its ability to establish causality definitively. Although multivariate adjustments and subgroup analyses were performed, residual confounding factors may still affect the observed outcomes. Second, we did not stratify the analyses by disease severity (e.g., GOLD stages) or clinical status (stable vs. acute exacerbation) due to database constraints. Due to the lack of stage-specific and exacerbation status data in the MIMIC-IV database, we could not perform subgroup analyses based on these important clinical factors. This limitation may impact the generalizability of our findings, as SII might behave differently in patients at different stages of COPD or during acute exacerbations. Future studies exploring SII in the context of COPD severity and exacerbation status are needed to elucidate its clinical utility and prognostic role. Third, our study calculated the SII from baseline neutrophil, platelet, and lymphocyte count measurements. Dynamic changes in the SII index during hospitalization or ICU stay were unavailable. Fourth, due to database constraints, the specific cause of death for each patient could not be determined, preventing the examination of SII’s role in cause-specific mortality among COPD patients. Finally, given this study’s single-center, retrospective design, our findings are limited in their generalizability. Multicenter prospective studies are required to validate these findings.

## 5 Conclusion

In conclusion, our study demonstrates that elevated serum immune-inflammation index (SII) levels are independently associated with increased mortality in critically ill patients with COPD. These findings support the potential of SII as a reliable prognostic biomarker for predicting mortality in this patient population. Incorporating SII into clinical practice may enhance risk stratification, enabling more informed decision-making and the development of personalized management strategies to improve patient outcomes.

## Data Availability

Publicly available datasets were analyzed in this study. This data can be found here: https://physionet.org/content/mimiciv/2.2/.

## Footnotes

^1^ https://github.com/MIT-LCP/mimic-iv
