# Supplementary material for: Association between systemic immune-inflammation index and mortality in critically ill patients with chronic obstructive pulmonary disease: insights from the MIMIC-IV database
Source: Front Med (Lausanne). 2025 May 16;12:1536652. doi: 10.3389/fmed.2025.1536652 (PMC12122298; doi:10.3389/fmed.2025.1536652)
Supplement: Supplementary file 1 [file Table_1.docx]

**Association between systemic immune-inﬂammation index and mortality in critically ill patients with Chronic Obstructive Pulmonary Disease: insights from the MIMIC-IV database**

Mohan Giri^1^, Anju Puri^2^, Lan Huang^1^, Shuliang Guo^1^*

^1^Department of Respiratory and Critical Care Medicine, The First Affiliated Hospital of Chongqing Medical University, Chongqing, China

^2^Department of Nursing, The First Affiliated Hospital of Chongqing Medical University, Chongqing, China

**Supplementary Table 1**: International Classification of Diseases (ICD) codes used to identify patients with Chronic Obstructive Pulmonary Disease.

| **ICD-9-CM codes** | **ICD-10-CM codes** |
| --- | --- |
| 49120, 49121, 49122, 496, 4910, 4920, 4928, 4919 | J440, J441, J449, J439 |

**Supplementary Table 2.** Univariate Cox Regression analysis for factors affecting in-hospital mortality in the study population.

| **Variable** | **HR (95% CI)** | **P-Value** |
| --- | --- | --- |
| Age | 1.032 (1.023–1.040) | < 0.001 |
| Gender (Male) | 0.924 (0.775–1.103) | 0.382 |
| SII | 1.165 (1.081–1.255) | < 0.001 |
| CCI | 1.113 (1.080–1.147) | < 0.001 |
| SOFA | 1.151 (1.128–1.176) | < 0.001 |
| SAPSII | 1.045 (1.039–1.050) | < 0.001 |
| OASIS | 1.076 (1.066–1.087) | < 0.001 |
| Hemoglobin | 0.980 (0.940–1.022) | 0.357 |
| WBC | 1.005 (1.001–1.009) | 0.011 |
| RBC | 0.947 (0.838–1.070) | 0.383 |
| Platelets | 0.999 (0.999–1.000) | 0.191 |
| Neutrophils | 1.024 (1.014–1.035) | < 0.001 |
| Lymphocytes | 1.000 (0.991–1.009) | 0.950 |
| Glucose | 1.002 (1.001–1.003) | < 0.001 |
| Creatinine | 1.108 (1.051–1.167) | < 0.001 |
| BUN | 1.012 (1.009–1.015) | < 0.001 |
| Bicarbonate | 0.950 (0.933–0.969) | < 0.001 |
| INR | 1.200 (1.132–1.272) | < 0.001 |
| APTT | 1.011 (1.007–1.015) | < 0.001 |
| Hypertension (Yes) | 0.810 (0.673–0.975) | 0.026 |
| Diabetes (Yes) | 0.795 (0.656–0.965) | 0.02 |
| Congestive Heart Failure (Yes) | 1.022 (0.856–1.220) | 0.81 |
| CAD (Yes) | 1.275 (1.062–1.530) | 0.009 |
| Renal Disease (Yes) | 1.117 (0.919–1.358) | 0.267 |
| Malignant Cancer (Yes) | 1.400 (1.131–1.732) | 0.002 |
| Severe Liver Disease (Yes) | 1.787 (1.287–2.482) | 0.0005 |
| Cerebrovascular Disease (Yes) | 1.250 (0.999–1.564) | 0.051 |
| Heart Rate | 1.012 (1.007–1.018) | < 0.001 |
| MAP | 0.981 (0.972–0.991) | < 0.001 |
| Respiratory Rate | 1.085 (1.063–1.108) | < 0.001 |
| SpO2 | 0.924 (0.894–0.955) | < 0.001 |
| Mechanical Ventilation (Yes) | 1.701 (1.415–2.045) | < 0.001 |
| Diuretic (Yes) | 0.954 (0.782–1.162) | 0.638 |
| RRT (Yes) | 1.675 (1.315–2.132) | < 0.001 |
| LOS ICU | 0.983 (0.971–0.995) | 0.004 |

APTT: Activated Partial Thromboplastin Time; BUN: Blood Urea Nitrogen; CCI: Charlson Comorbidity Index; CAD: Coronary Artery Disease; INR: International Normalized Ratio; LOS ICU: Length of Stay in the Intensive Care Unit; MAP: Mean Arterial Pressure; RBC: Red Blood Cell count; RRT: Renal Replacement Therapy; SII: Systemic Immune-Inflammation Index; SOFA: Sequential Organ Failure Assessment; SAPSII: Simplified Acute Physiology Score II; SpO2: Peripheral capillary oxygen saturation; WBC: White Blood Cell count.
